# Multi-Analytical Assessment of Deterioration in the Qianlong Tripitaka Wooden Scripture Plates

**DOI:** 10.3390/polym17212855

**Published:** 2025-10-26

**Authors:** Wangting Wu, Yuhan Peng, Jianrui Zha, Ge Zhang, Mengdie Lv, Yingzhu Wang

**Affiliations:** 1 Conservation Department, Capital Museum, Beijing 100045, China; wangtingwu@126.com (W.W.); am_xiaoga@163.com (G.Z.); lvmengdie86@126.com (M.L.); wangyingzhu88@163.com (Y.W.); 2 College of Applied Arts and Science of Beijing Union University, Beijing 100101, China; jane_pyh@163.com; 3 Key Laboratory of Archaeomaterials and Conservation, Ministry of Education, Institute of Cultural Heritage and History of Science and Technology, University of Science and Technology Beijing, Beijing 100083, China

**Keywords:** Qianlong Tripitaka, wooden cultural heritage, multi-analytical assessment, deterioration

## Abstract

The Qianlong Tripitaka preserved in the Capital Museum is a distinctive large-scale wood block printing plates of the Qing Dynasty. It represents a unique type of Chinese documentary wooden heritage preserved in a dry museum environment, which has rarely been subjected to comprehensive physicochemical analysis, resulting in an inadequate understanding of their deterioration processes. This study applied a comprehensive multi-analytical method to investigate the deterioration of the scripture plates. The findings indicate that the Qianlong Tripitaka shows typical structural deformation, chemical depolymerization, and a decline in structural integrity and stability. Scanning Electron Microscopy (SEM) and Computed Tomography (CT) revealed thinning and the distortion of cell walls, reduced density, and partial collapse of tissue structures. Thermogravimetric Analysis (TGA) indicated lower decomposition temperatures and higher inorganic residues, while a Brunauer–Emmett–Teller surface area analyzer (BET) showed diminished surface area, expanded pores, and compromised connectivity. Moisture content analyses verified significant water loss, contributing to brittleness and susceptibility to microbial degradation. Fourier Transform Infrared Spectroscopy (FTIR) and X-ray Diffraction (XRD) analyses revealed considerable hemicellulose degradation, the disruption of cellulose crystallinity, and relatively stable lignin. This study highlights the value of a multi-analytical strategy for assessing the deterioration of wooden cultural heritage, providing a transferable framework for similar documentary wooden artifacts.

## 1. Introduction

The Qianlong Edition Tripitaka is the officially carved Tripitaka in the Chinese language. Designated by Emperor Yongzheng of the Qing Dynasty, it was inscribed with the Ming Dynasty Northern Tripitaka as the primary reference with several modifications. The carving commenced in the 11th year of the Yongzheng reign of the Qing Dynasty (1733) and was completed in the 3rd year of the Qianlong reign (1738). The wooden relic is crucial for the study of block printing and the evolution of Buddhism. The scripture plates are constructed from pear wood, measuring 75 cm in length, 35 cm in width, 5 cm in thickness, and weighing around 4.5 kg, exhibiting a thin rectangular form. After completion of the scripture plates carving, the scripture plates were significantly damaged due to inadequate preservation resulting from changes in management authority, warehouse renovations, and frequent transportation. To date, only 69,410 carved plates of the Qianlong Edition Tripitaka are preserved in the Capital Museum, Yunju Temple in the Fangshan District, and the Zhihua Temple, with 9626 plates having been lost. Among the existing scripture plates, 20% have suffered varying degrees of damage. Its preservation and restoration require a thorough understanding of its deterioration.

Previous research has investigated the deterioration mechanisms of wooden cultural relics from both environmental and material perspectives. Blanchette et al. conducted a systematic analysis on the microbial degradation of archeological wood, revealing that white rot, brown rot, soft rot fungi, and other bacterial types exhibit distinct mechanisms of cell wall deterioration modes under dry, moist, and submerged conditions [1]. Tamburini et al. analyzed 11 archeological wood samples from arid Egyptian burials employing SEM, FTIR, wet chemistry, and Py-GC/MS, revealing diverse degradation routes characterized by the preferential or simultaneous loss of polysaccharides and lignin [2]. Ghavidel et al. utilized XPS, XRD, and DVS to analyze excavated elm and poplar, revealing surface depolymerization/oxidation, reduced crystallinity, and enhanced hygroscopic behavior compared to fresh wood [3]. Singh et al. completed a review of microbial research on buried and waterlogged woods, highlighting soft rot fungi and erosive bacteria as dominant agents, with the latter being more prevalent in anoxic environments [4]. Popescu et al. applied XPS to analyze aged linden panels (6–250 years), showing initial oxidation and hydrolysis, with a preferential degradation of hemicellulose and amorphous cellulose, while crystalline cellulose and lignin remained relatively stable [5]. Beyer et al. combined wet chemistry, Raman, and GC/MS to study naturally aged spruce, fir, and oak, finding initial extractive loss, pronounced hemicellulose reduction, mild lignin oxidation, and gradual cellulose crystallinity decline depending on the species and environment [6].

Wooden objects in the museum are highly susceptible to destruction by the microbial community. Pyzik et al. indicated that fungi and bacteria accelerate the degradation of cellulose, hemicellulose, and lignin through extracellular enzymes, biofilm formation, and metabolic byproducts, thereby reducing mechanical strength and aesthetic integrity, while certain strains have been explored for bioremediation strategies, such as salt removal or carbonate reinforcement [7]. Liu et al. proposed a diagnosis, mechanism, and management framework that integrates non-destructive survey, targeted sampling, high-throughput sequencing, and metagenomics to correlate the microbial community structure with deterioration processes [8]. Afifi et al. investigated a pinewood panel decoration with plaster, paint, and gilding from the tomb of Sultan Qutb al-Din in Cairo through the SEM-EDX, XRD, and FTIR technique, identifying *Aspergillus niger*, *A. flavus*, and *A. terreus* as predominant fungi, with simulated tests confirming that *A. niger* and *A. terreus* induced pigment discoloration and gypsum degradation [9]. Geweely et al. investigated three ancient Egyptian wooden artifacts through OM, SEM, FTIR, and synchrotron XRD, revealing multi-coupled deterioration features such as microcracks, salt crystallization, cell wall separation, fungal infection, reduced cellulose crystallinity, and relative lignin enrichment [10]. In a related study, Afifi et al. assessed an Ottoman stained-glass inlaid plaster window at the Bab Al-Azab district, combining digital microscopy, PLM, OM, SEM-EDX, XRD, and FTIR, which revealed gypsum, calcite, anhydrite plaster with salt impurities, pinewood with a fungal infestation and reduced cellulose crystallinity, and glass containing transition metal-based coloring oxides [11].

To better understand the preservation state of wooden artifacts, scholars have developed diverse analytical and diagnostic methods. Łucejko et al. presented OM, SEM, UV/IR, Raman, NMR, and pyrolysis-based MS/GC for archeological wood, demonstrating the preferential degradation of cellulose and hemicellulose and the relative enrichment of lignin [12]. Gelbrich et al. applied FTIR spectroscopy to diagnose bacterial decay in historic wood, revealing increased lignin absorption bands that exhibited a strong correlation with microscopic and chemical analyses, enabling rapid non-destructive regression models for lignin quantification and deterioration assessment [13]. Pizzo et al. combined ATR-FTIR with PLS modeling on 59 waterlogged wood samples from various species and environments, illustrating that calibration must consider lignin, holocellulose, ash, and wood type [14]. Traoré et al. conducted a comparative analysis of cathedral beams and shipwreck wood via FTIR-ATR with PCA and Py-GC–MS. Their findings indicated that pine beams retained a greater quantity of polysaccharides and exhibited reduced lignin content, while oak shipwreck wood showed marked carbohydrate loss and lignin enrichment [15]. Boukir et al. studied Moroccan argan wood using ATR-FTIR and XRD, demonstrating the loss of the hemicellulose peak, weakening of lignin, formation of quinone, cleavage of the C–O–C bond, and a diminished crystallinity index, thereby confirming progressive microfibril amorphization [16]. Huang et al. investigated ancient Chinese cedar coffins through microscopy, chemical composition, GPC, and 2D NMR, showing significant polysaccharide loss, regional lignin depolymerization at cell corners, the formation of new hydroxyl groups, and late-stage condensation, indicating both oxidative depolymerization and partial crosslinking [17]. High K/E values demonstrate that the wood remains significantly fragile due to multifactorial degradation, highlighting the necessity for comprehensive multi-analytical strategies from macroscopic to molecular scales [18].

In recent years, multi-technique combined analysis has become crucial for assessing wooden cultural relics. Haneca et al. applied sub-micron X-ray CT to mineralized wood from the Broechem cemetery, achieving genus/species-level identification and virtual sectioning, evaluating the mineralization for conservation strategies [19]. Stelzner and Million used industrial microfocus and sub-micron CT on medieval wood from Lauchheim, facilitating species identification, tree-ring analysis, and dating, while noting the resolution, sample size, wood type, and preservation condition as limiting factors [20]. Daly and Streeton validated non-invasive dendrochronology for medieval reliquaries, altarpieces, and sculptures in Oslo, combining the digital imaging of growth rings with industrial CT, which revealed rings beneath paint, gilding, and composite structures, supporting chronological dating studies [21]. Ghavidel et al. conducted a comparative analysis of fresh and 150–200-year-aged spruce and fir using ATR-FTIR, XPS, and XRD, revealing hemicellulose degradation in fir, pronounced cellulose/hemicellulose decay in spruce, surface oxidation and hydrolysis, as indicated by XPS, and divergent crystallinity trends under indoor and outdoor exposure [22]. Longo et al. pioneered the measurement of multislice clinical CT density coupled with FTIR and Raman techniques, demonstrating complementary macro-density and micro-chemical analysis [23]. Atwa et al. studied wood from Khufu’s Second Solar Boat using SEM-EDX, XRD, FTIR, FISH, and PCR, revealing collapsed cell walls, salt penetration, reduced crystallinity, relative lignin enrichment, and the predominance of Aspergillus and Penicillium [24]. Abdrabou et al. performed a multi-technique analysis of Middle Kingdom coffins, validating the use of Ficus sycomorus for panels and Tamarix for joints, while identifying pigments, such as glauconite, optical blue, and ochre [25]. Čufar et al. identified Bambuti wooden artifacts through minimally invasive microanatomy, corroborated by the InsideWood database and Tervuren collections, confirming species such as *Autranella congolensis*, *Nauclea diderrichii*, and *Xylopia* sp. [26]. Vigorelli et al. analyzed a Middle Kingdom wooden statue with a two-stage strategy, UVF, VIL, IRR, RX, and CT imaging, followed by XRF, OM, FTIR, and SEM-EDX, revealing mortise and tenon joints, CaCO_3_ ground layers, ochre and carbon black pigments, and Paraloid repairs [27]. Bossema et al. developed an economical method for reconstructing 3D CT from conventional 2D X-rays with metal markers and algorithms, successfully applied at the British Museum, Getty, and Rijksmuseum, offering safe, accessible internal visualization [28]. Dierickx et al. evaluated μ-CT across 17 African wood species at resolutions of 1–15 µm, finding that 3 µm was optimal for balancing detail and the field of view, while 1 µm revealed fiber wall thicknesses, and greater scales recorded significant features [29]. In another case, they used μ-CT on 20 sub-Saharan artifacts from the Royal Museum for Central Africa, identifying 22 specimens and correlating wood properties with artifact functions, such as lightweight woods for ceremonial use and dense woods for acoustic instruments [30]. Ibrahim et al. analyzed Khufu’s Second Solar Boat using synchrotron micro-CT, SEM, XRD, FTIR, and molecular techniques, confirming the presence of Cedrus libani timber, cellular collapse, mineral infiltration, reduced cellulose crystallinity, relative lignin enrichment, and an infestation of Aspergillus/Penicillium, predominantly featuring cellulolytic *A. flavus* and *A. terreus* [31].

Despite several studies investigating the deterioration of wooden relics in buried or waterlogged conditions through multi-analytical approaches, research on large-scale documentary wooden heritage preserved in dry museum environments is still limited. The Qianlong Tripitaka serves as an important case to address this deficiency. This study integrates OM, SEM, CT, FTIR, XRD, BET, and DVS to establish a comprehensive deterioration profile for dry stored documentary wooden artifacts.

## 2. Materials and Methods

### 2.1. Materials

#### 2.1.1. Qianlong Tripitaka

**Figure 1:**
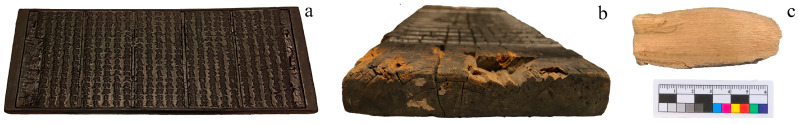
Image of Qianlong Tripitaka (**a**), surface section (**b**), and cross section (**c**) sample and color charts.

Figure 1 illustrates the image of Qianlong Tripitaka. The Qianlong Tripitaka shows a rectangular, thin-plate form. Surfaces (Figure 1a) exhibit Buddhist scriptures inscribed in regular script. The surface is primarily flat, although it displays varied types of deterioration. The cross section (Figure 1b) indicates that the scripture plate contains a porous internal structure. Such deterioration includes fissures, degradation, and discoloration. The experimental sample (Figure 1c) was obtained from the scripture plate edge; sample size is 8.2 cm × 3.3 cm × 1.2 cm, and it was stored in the laboratory at approximately 60 RH% and 20 °C.

#### 2.1.2. Pear Wood

**Figure 2:**
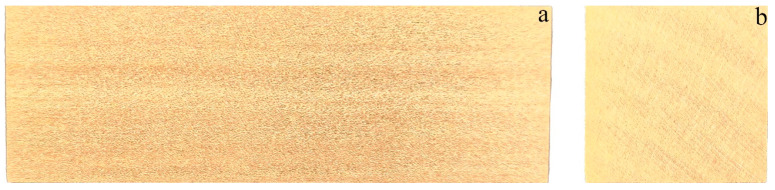
Image of pear wood (**a**) surface section; (**b**) cross section.

Figure 2 shows an image of pear wood; the samples were obtained from Zhongding Cultural Development Co., Ltd. (Jinan, China). According to standard GB/T 15777-2017, method for determination of the modulus of elasticity in compression parallel to grain of wood, publisher: Beijign, China, 2017 [32], sample was cut to 2 cm × 2 cm × 6 cm size for testing. In Figure 2a, straight and parallel longitudinal grain lines are distinctly observed, signifying dense and well-aligned fibers. The surface is smooth and displays a natural luster, reflecting the fine texture and homogeneous structure of pear wood. Figure 2b shows the cross section of pear wood, which clearly exhibits annual rings arranged in a circular structure containing alternating earlywood and latewood bands with pronounced light and dark coloration.

### 2.2. Analysis Method

#### 2.2.1. OM

A KH-8700 ultra-depth-of-field 3D microscope (Haoshi, China) was used to observe the morphology of the samples. The magnification used for observation is 200×.

#### 2.2.2. SEM

The longitudes and cross sections of the samples were prepared using a pathological knife, affixed on the sample stage with conductive glue, and coated with gold via an ion sputtering apparatus. The gold-coated scriptural plate samples were examined using a S-3400N Scanning Electron Microscope (Hitachi, Japan) at a working voltage of 15 kV. High-vacuum conditions were used.

#### 2.2.3. FTIR

IRPrestige-21 Fourier Transform Infrared Spectroscopy (Shimadzu, Japan) with ATR (Attenuated Total Reflection) was used for testing. The scanning wave number ranged from 4000 cm^−1^ to 750 cm^−1^, the number of scans was 20, and the resolution was 4 cm^−1^. Solid samples were tested directly.

#### 2.2.4. XRD

The samples were tested in an XPertPro MPD X-ray diffractometer (Malvern Panalytical, Holland) at room temperature. A copper target X-ray tube was used with Cu Kα radiation (λ = 1.5406 Å) with a voltage of 40 kV and a current of 40 mA. The scanning angle range was 5° to 45°, the scanning speed was 0.03°/s, and the step size was 0.0021°. The standard for phase identification was PDF-2 database, ICDD standard [33].

#### 2.2.5. CT

A Comet Yxlon FF85 CT was used for analysis under the following conditions: continuous scanning with a conical beam, a 300 kV microfocus X-ray tube, a tube voltage of 240 kV, a tube current of 280 μA, a flat panel detector mode of 1 × 1 binning mode (0.5 pF) detector capacitance, exposure time was 285.714 ms, number of projections was 2000, and a spatial resolution of 50 μm. We used the tested sample to calibrate the detector in order to search for the appropriate voltage and current. The software used for reconstruction was VGStudio MAX 2025.2.

#### 2.2.6. TGA

A Q5000 thermogravimetric analyzer (TA Instruments, New Castle, DE, USA) was used. The mass of the pear wood sample was 0.00755 g, the mass of the scripture plate sample was 0.00691 g. The test temperature was raised to 800 °C at a heating rate of 10 °C·min^−1^, and the test was conducted under a nitrogen atmosphere. Degassing temperature was 100 °C; duration was 7.5 h.

#### 2.2.7. BET

A Micromeritics ASAP2460 pore size analyzer was used to test the samples. The mass of the pear wood sample was 0.9756 g, the mass of the scripture plate sample was 1.1860 g. According to GB/T 19587-2017 [34], nitrogen (N_2_) was used as the adsorptive gas at a bath temperature of −195.8 °C. The BET surface area was calculated in the relative pressure (P/P_0_) range of 0.05–0.35 for linear fit.

## 3. Results

**Figure 3:**
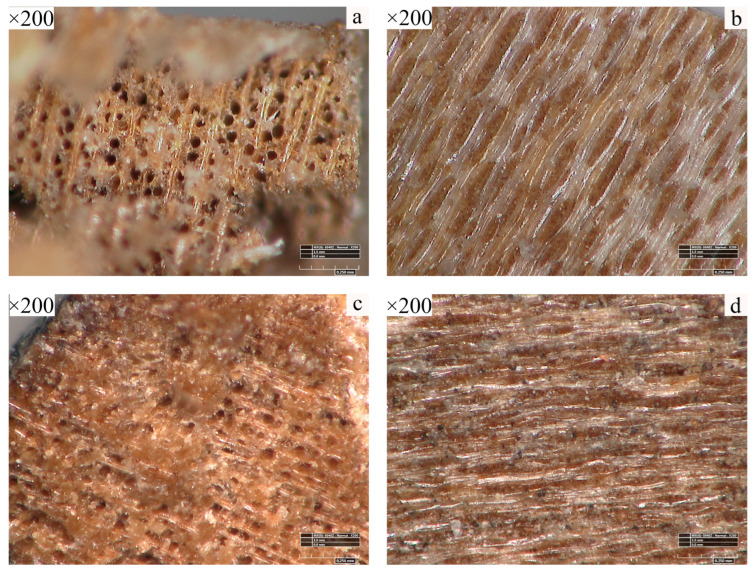
Microscope image. (**a**) Cross section of pear wood, (**b**) longitudinal section of pear wood. (**c**) Cross section of Qianlong Tripitaka, (**d**) longitudinal section of Qianlong Tripitaka.

Figure 3 presents microscopic images (×200) of pear wood (a, b) and the scripture plate (c, d). The cross section of pear wood (a) exhibits uniformly distributed circular vessel lumens, accompanied by distinct rays and dense fiber tissue. The longitudinal section (b) displays neatly arranged fibers, continuous rays with distinct boundaries, intact cell walls, and a smooth surface. In contrast, the scripture plate exhibits pronounced aging and degradation. The cross section (c) shows irregularly shaped vessels with partial collapse filled with substances, while the boundaries between fibers and rays remain ambiguous. The longitudinal section (d) reveals disordered fiber arrangement, overall darkened cell walls, and a looser structure.

**Figure 4:**
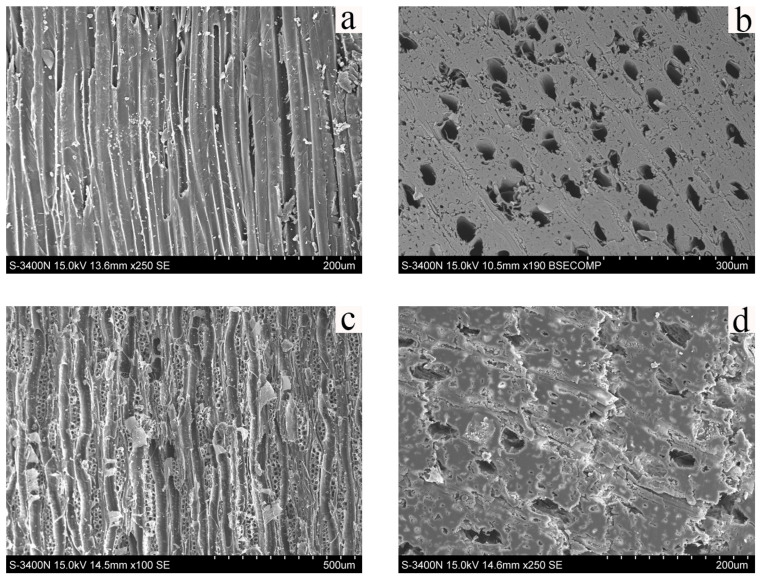
SEM images of sample (**a**) longitudinal section of pear wood, (**b**) cross section of pear wood, (**c**) longitudinal section of scripture plate, and (**d**) cross section of scripture plate.

Figure 4 displays the SEM images of pear wood and the scripture plate. In the longitudinal section of pear wood (Figure 4a), the cell walls have a smooth, thick, and continuous appearance, signifying excellent structural integrity. The cross section of pear wood (Figure 4b) has a dense and uniform microstructure. In the longitudinal section of the scripture plate (Figure 4c), the middle lamella has largely disappeared, the secondary cell walls appear thinned and locally deformed, and several cells display partial delamination and peeling. The cross section of the scripture plate (Figure 4d) exhibits irregular vessel lumina, with some cells enlarged, collapsed, or filled with degradation products. Compared with the structure of pear wood, the scripture plate shows significant degradation, marked by middle lamella loss, the thinning and distortion of the cell walls, irregular lumen morphology, and diminished structural support capacity.

**Figure 5:**
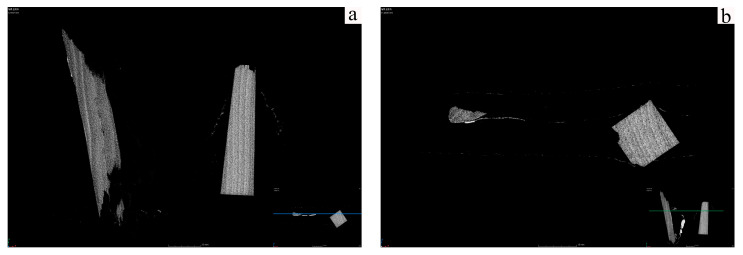
CT images of sample (**a**) transverse section (The blue line indicates the position of the CT slice), (**b**) longitudinal section (the green line indicates the position of the CT slice).

On the longitudinal section (Figure 5b), the two-dimensional pore area fraction in the pear wood was approximately 47%, while the scripture plate wood reached as high as 66%. Compared to transverse sectional (Figure 5a) results (about 38% for archeological wood and 21% for fresh wood), the longitudinal section exhibits higher porosity, indicating the presence of multiple continuous vessels and fissures aligned with the fiber direction. The significant increase in longitudinal porosity of archeological wood suggests that cell wall deformation creates continuous pathways, leading to a mechanical strength decrease.

**Figure 6:**
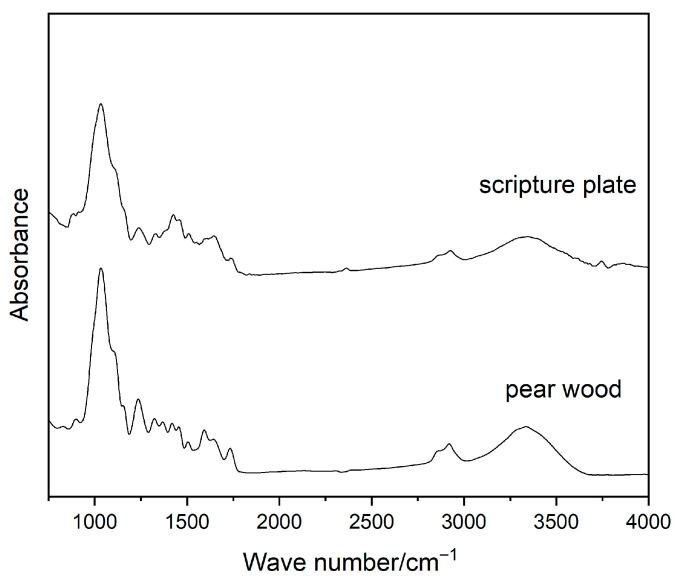
Infrared spectra of sample (**a**) pear wood, (**b**) scripture plate.

The infrared spectra of the pear wood and scripture plate are shown in Figure 6. The absorption peak at 1732 cm^−1^ is attributed to the C=O stretching vibration of the acetyl group and hydroxyl group in the hemicellulose molecule. Compared with the pear wood, the intensity of this peak in the scripture plate decreases and tends to disappear, which indicates that the number of C=O groups decreases, and hemicellulose degradation occurs. The peak at 1595 cm^−1^ is attributed to the C=O stretching vibration of lignin, and the peak near 1505 cm^−1^ is C=C aromatic skeletal vibration in lignin [35]. The degradation of lignin is affected by the subsequent degradation of hemicellulose. The peak at 1368 cm^−1^ is ascribed to holocellulose. The significant decrease in the peak intensity of the scripture plate at this position indicates a decline in the C-H stretching vibration and the C-O-C stretching vibration in cellulose. The infrared spectrum of the scripture plate reveals well-preserved absorption at 1235 cm^−1^ and 1035 cm^−1^, indicating that the large lignin molecules in the scripture plate have not undergone considerable degradation. The peak at 895 cm^−1^ is ascribed to the C-H vibration of cellulose. The peak position shifts and the peak intensity almost disappears, indicating a partial disruption of the hydrogen bond network in the cellulose crystals of the scripture plate, resulting in a significant decrease in the peak intensity and damage to the cellulose crystalline region [36]. This indicates that the hemicellulose in the scripture plate exhibits significant degradation.

**Figure 7:**
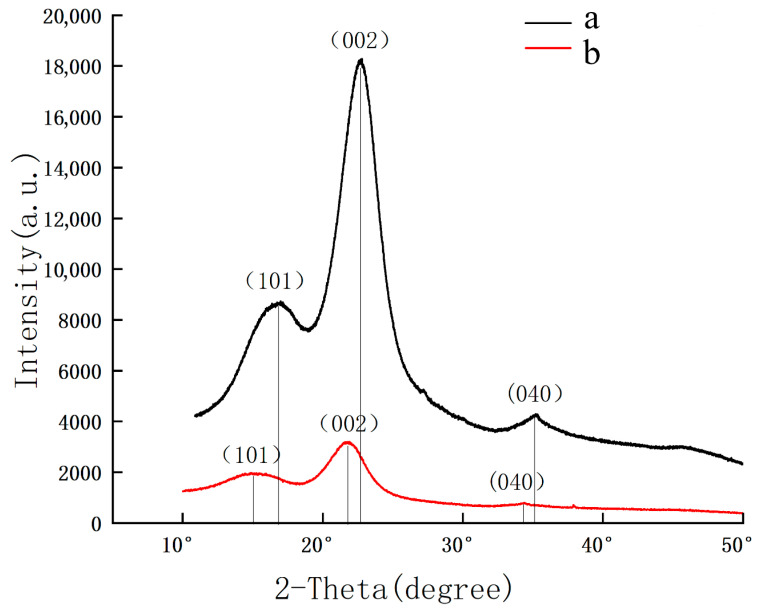
XRD patterns of sample (**a**) pear wood, (**b**) scripture plate.

X-ray diffraction tests were conducted on pear wood and the scripture plate, and the results are shown in Figure 7. The diffraction peak area of the pear wood is higher than that of the scripture plate. The 2θ angles are located near 16°, 22.5°, and 35°, corresponding to the crystal planes of (101), (002), and (040), respectively. Moreover, the peak angles of the scripture plate are lower than those of the pear wood. The intensities of the diffraction peaks for the (101) and (002) crystal planes decrease with the aging of the wood. The diffraction peak of the (040) crystal plane tends to disappear due to degradation, indicating the gradual dissociation of the cellulose crystal. The Segal empirical method [37] was used to calculate the crystallinity of the wood cellulose. According to the crystallinity formula CrI (%) = [(I002 − I101)/I002] × 100%, the crystallinity of the pear wood was calculated to be 53%, and that of the scripture plate wood was 49%. The crystallinity results indicate that the crystalline region of the scripture plate is separated and dispersed, signifying the deterioration of cellulose and macromolecules [38].

**Figure 8:**
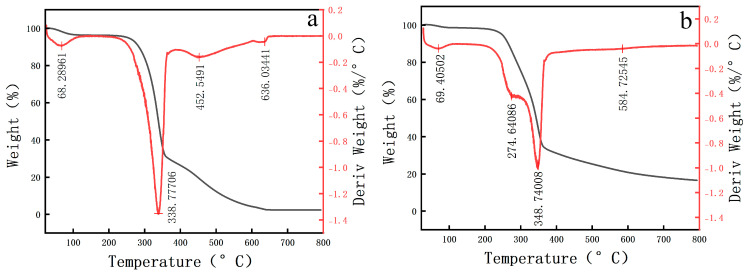
DTA-TG results of sample (**a**) pear wood, (**b**) scripture plate.

The DTA-TG results of the pear wood and scripture plate are shown in Table 1 and Figure 8. The thermal weight loss of both the pear wood and scripture plate can be divided into four distinct stages. The initial stage includes water loss, followed by the main decomposition stage, which corresponds to the decomposition of hemicellulose (220–315 °C) and cellulose (315–400 °C). The secondary decomposition stage (400–500 °C) focuses on the degradation of lignin, while the high temperature stable stage consists of the residual inorganic materials. Compared with the pear wood, the temperatures corresponding to the last three stages of scripture plate are lower, indicating that the macromolecular compounds of the scripture plate are more likely to decompose. In the initial stage, the weight loss rate of the scripture plate is lower than that of the pear wood, which proves that the moisture content of the scripture plate wood is relatively low. During the long-term preservation process, the free water molecules in the wood have evaporated and been lost, and the holocellulose has been partially decomposed.

**Figure 9:**
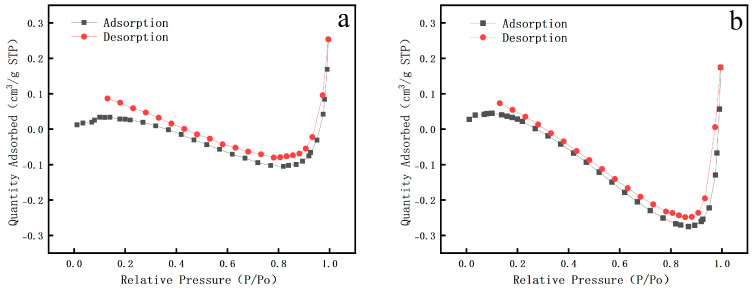
BET results of sample (**a**) pear wood, (**b**) scripture plate wood.

The BET test results (Figure 9) indicate that the nitrogen adsorption isotherms for both the scripture plate and pear wood are classified as Type IV, with H2-type hysteresis loops. Pear wood is a mesoporous material, and its adsorption capacity increases significantly in the medium to high pressure range. As the relative pressure (P/Po) markedly rises from around 0.4, the maximum adsorption capacity can attain 0.3 cm^3^/g (STP), and the hysteresis loop ends with a lag. The scripture plate has similar characteristics, but its adsorption capacity is lower. Within the same relative pressure range, its adsorption capacity is lower than that of pear wood, with a maximum of 0.25 cm^3^/g (STP), and the hysteresis loop has a larger span. Table 2 illustrates that the specific surface area of the scripture plate is significantly lower than that of pear wood (exhibiting a reduction of 10–15%). At the same time, the pore size of pear wood is smaller, and its distribution is more uniform, while the average pore size of the scripture plate wood is significantly larger.

## 4. Discussion

In contrast with previous studies on wood relics buried or waterlogged, the Qianlong Tripitaka Wooden Scripture Plates, preserved in dry, museum-like environments, exhibit a distinct degradation pathway. They experience significant water loss leading to shrinkage, cracking, and brittleness instead of maintaining a plasticized state. In water-saturated environments, microbial enzymatic activity dominates the decay. Upon exposure, the transition from anaerobic to aerobic conditions further accelerates polysaccharide hydrolysis, and the oxidation of iron sulfides can acidify the wood, resulting in significant hemicellulose loss, diminished cellulose crystallinity, and a polysaccharide degradation [39].

By contrast, in dry-preserved conditions, the decay of archeological woods occurs mostly through gradual oxidative and hydrolytic reactions rather than microbiological activity. Dellaportas et al. highlighted that, in museum environments, organic heritage materials are predominantly affected by abiotic factors, such as temperature, relative humidity fluctuations, airborne particulates, and gaseous pollutants. These stressors promote gradual chemical aging and oxidation processes, even in the absence of biological activity [40]. Davis et al. conducted an in situ conservation and analytical study of severely deteriorated, dry painted wooden statues from Abydos, Egypt, where the substrate exhibited a loss of structural cohesion and primarily comprised degraded wood parts. Their findings indicated that deterioration was primarily driven by oxidation, gradual hydrolysis, and the progressive disintegration of cellulose and hemicellulose networks [41].

In our study, hemicellulose experiences considerable hydrolysis, and cellulose undergoes partial amorphization, primarily driven by slow oxidation and hydrolysis in the long-term dry environment rather than microbial enzymatic degradation. Lignin remains intact with mild oxidative modification, in contrast to the substantial lignin enrichment observed in waterlogged wood resulting from massive polysaccharide loss. Structural collapse observed via CT predominantly results from drying shrinkage and gradual chemical degradation. The modification of the pore structure and surface contamination suggests that the inorganic compounds originate more from long-term dust deposition than groundwater salt infiltration.

This distinction indicates that preventive conservation for large woodblock-printed plates preserved in dry environments should emphasize humidity regulation and the mechanical reinforcement of cellulose/hemicellulose, alongside surface contamination management. In our previous research, we attempted to prepare a humidity-responsive epoxy resin and achieved satisfactory results [42]. This approach differs from waterlogged wooden relics, where disinfection, mold control, and desalination are primary interventions.

## 5. Conclusions

This work reveals that the Qianlong Tripitaka wooden scripture plates, after long-term dry storage, exhibit cell wall thinning and collapse, significant hemicellulose hydrolysis, partial cellulose amorphization, and relatively stable but mildly oxidized lignin. In the scripture plates, increased longitudinal porosity and reduced crystallinity compromise mechanical integrity, while BET and CT show larger, irregular pores and lower specific surface area. In contrast to waterlogged wood, deterioration in the scripture plate is mostly caused by moisture loss and gradual oxidation rather than microbial decay. Conservation should emphasize humidity regulation, cellulose/hemicellulose reinforcement, and surface contamination control.

## Figures and Tables

**Table 1:** DTA-TG results of samples.

Decomposition Stage		Initial Weight Loss Water Evaporation	Main Decomposition Stage Cellulose/Hemicellulose Decomposition	Secondary Decomposition Stage Lignin Decomposition	High-Temperature Stable Stage Inorganic Residues
Pear wood	Temperature (°C)	68.28	338.77	452.54	636.03
	Weight Loss Rate (%)	3.767	69.015	23.207	1.720
Scripture Plate Wood	Temperature (°C)	69.40	274.64	348.74	584.72
	Weight Loss Rate (%)	1.71	13.886	55.459	16.547

**Table 2:** BET data table of scripture plate wood and pear wood.

Type	Specific Surface Area	Average Pore Size
Pear wood	415–420 m^2^/g	4.1–4.2 nm
Scripture Plate Wood	365–380 m^2^/g	6.0–6.3 nm

## Data Availability

The raw data supporting the conclusions of this article will be made available by the authors on request.
